# Comparison of Model Predictions and Laboratory Observations of Transgene Frequencies in Continuously-Breeding Mosquito Populations

**DOI:** 10.3390/insects7040047

**Published:** 2016-09-22

**Authors:** Laura Valerio, Ace North, C. Matilda Collins, John D. Mumford, Luca Facchinelli, Roberta Spaccapelo, Mark Q. Benedict

**Affiliations:** 1 Department of Experimental Medicine, University of Perugia, Via Gambuli, Building D, 3rd floor, Perugia 06132, Italy; scarablaura@yahoo.it (L.V.); lucafacchinelli@yahoo.it (L.F.); roberta.spaccapelo@unipg.it (R.S.); 2 Department of Zoology, University of Oxford, The Tinbergen Building, Oxford OX1 3PS, UK; ace.north@zoo.ox.ac.uk; 3 Centre for Environmental Policy, Imperial College London, 14 Princes Gardens, London SW7 2NA, UK; t.collins@imperial.ac.uk; 4 Centre for Environmental Policy, Imperial College London, Silwood Park, Ascot SL5 7PY, UK; j.mumford@imperial.ac.uk

**Keywords:** GMO, transgenic mosquito, risk assessment, persistence, malaria, genetic control, sterile insect technique

## Abstract

The persistence of transgenes in the environment is a consideration in risk assessments of transgenic organisms. Combining mathematical models that predict the frequency of transgenes and experimental demonstrations can validate the model predictions, or can detect significant biological deviations that were neither apparent nor included as model parameters. In order to assess the correlation between predictions and observations, models were constructed to estimate the frequency of a transgene causing male sexual sterility in simulated populations of a malaria mosquito *Anopheles gambiae* that were seeded with transgenic females at various proportions. Concurrently, overlapping-generation laboratory populations similar to those being modeled were initialized with various starting transgene proportions, and the subsequent proportions of transgenic individuals in populations were determined weekly until the transgene disappeared. The specific transgene being tested contained a homing endonuclease gene expressed in testes, *I-Ppo*I, that cleaves the ribosomal DNA and results in complete male sexual sterility with no effect on female fertility. The transgene was observed to disappear more rapidly than the model predicted in all cases. The period before ovipositions that contained no transgenic progeny ranged from as little as three weeks after cage initiation to as long as 11 weeks.

## 1. Introduction

The persistence of a transgene in populations of genetically modified organisms in the environment is an important consideration in determining its risk [1,2], however, persistence is not, in itself, an indication of harm, which would be a consideration in risk assessment. Genetically-modified mosquitoes in some cases are already being used in the field [3,4] and, in other cases, are being considered for control of vector-borne diseases [5,6,7]. The transgenes may persist for varying lengths of time in populations in the environment, depending on the nature of the modification and its phenotype. Reliable prediction of such persistence is an important component of both risk and performance assessments.

Heritable factors (including transgenes) that spread and persist can be desirable for prolonged disease suppression [8,9,10], and these qualities are traits of particular types of transgenes that are currently being created [11,12]. In contrast, for those conferring sexual sterility [13] or larval lethality conferred by systems such as RIDL^TM^ [14], persistence is neither intended nor expected, due to the phenotype of the transgene. Such transgenes have been referred to as “self-limiting” [2], reflecting their expected disappearance from wild populations. An incremental step-by-step process of developing self-limiting approaches prior to persistent transgenes is recommended for development of genetically-modified mosquitoes [2], in part so that predictive tools can be devised and tested experimentally to ensure that the modified-mosquito behaviors expected actually occur or, if deviations from the models occur, whether these present greater risk than what was expected prior to the observations.

Experimental methods and examples for estimating “fitness” and likely persistence of transgenes in mosquito populations include life table analysis [15] and discrete generation population cage studies [16,17]. In at least one case, specific potentially selective influences have been applied during the cultivation of the mosquitoes that affected estimates of fitness, specifically the presence of malaria parasites in mosquitoes that were resistant to infections [18]. The method we utilize here, studies with overlapping generations in continuously maintained cage populations, offers yet another approach.

We modelled, using stochastic simulations, and in parallel, measured in laboratory experiments, the proportion of individuals carrying a transgene that causes complete sexual sterility in male hemizygotes. We seeded populations of mosquitoes with various proportions of transgenic individuals and measured the transgene frequency weekly among progeny in continuously maintained cages containing overlapping generations. No deliberate effort was made to select transgenic individuals.

The strains carry a transgene that encodes an endonuclease that recognizes and cuts the ribosomal DNA (rDNA) which, in *Anopheles gambiae*, is located mainly on the X chromosome [19] but, at least in some laboratory populations, is also found on the Y [20]. This causes cleavage and loss of the X chromosome in sperm. Available evidence [13,21] demonstrates that this form of the I-*Ppo*I nuclease persists in sperm and cuts the maternal X chromosome in the embryonic pronucleus, leading to failure of fertilized embryos to develop to hatching.

## 2. Materials and Methods

### 2.1. Modelling

**Figure 1:**
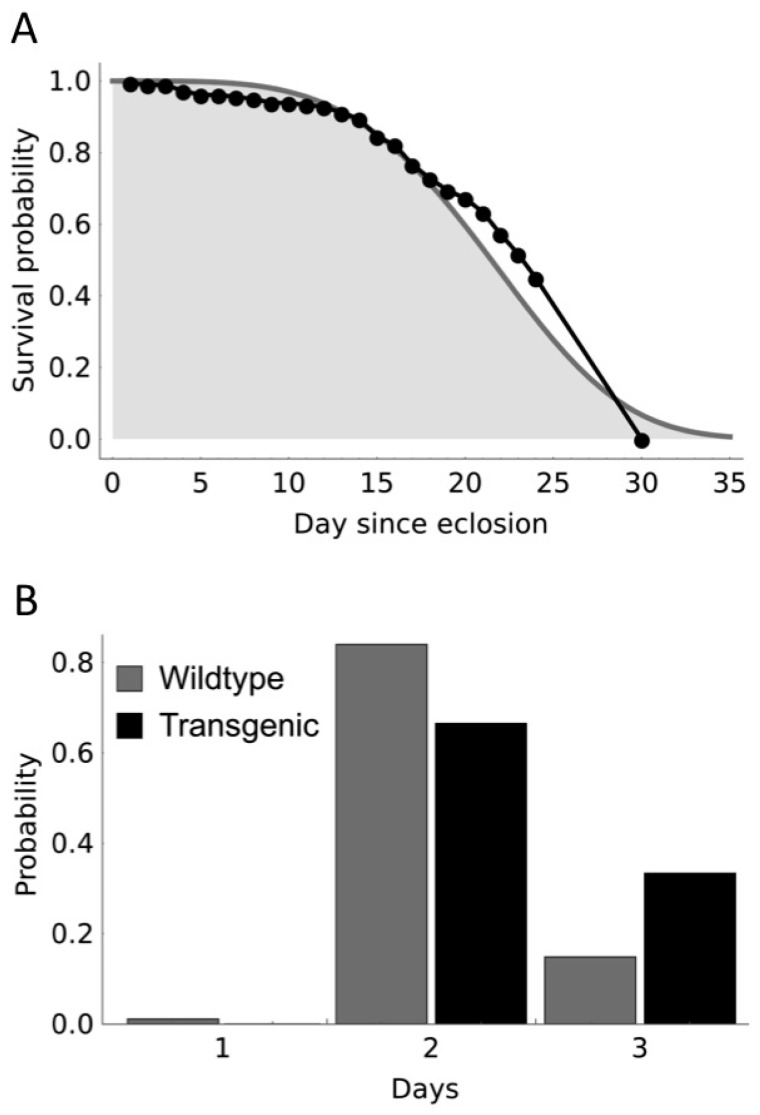
Adult longevity and duration of pupation used for the model. (**A**) illustrates adult longevity data (black markers) of [23] that was used to fit a Weibull curve for survival (solid line delimiting gray area fitted by maximizing likelihood). In addition to the data which was available for 24 days, we assumed all adults died before day 30; and (**B**) the distribution of pupal development times used for model parameterization is shown, based on summarized data [22].

We modelled the cage experiments by making a number of simplifying assumptions on the behavior of the genetically-modified (GM) and wild-type (WT) mosquitoes. First, no progeny are observed from matings of hemizygous transgenic males, as has been reported previously [13,22]. Second, the life span of each individual adult female (time in days from adult eclosion to death) is assumed to be Weibull-distributed. The shape and scale parameters of the Weibull distribution are fitted from published survival data [23], with k = 4.07 and lambda = 23.5 (Figure 1A). Third, females mate and feed within two days of emerging as adults, so that all females that are at least three days old are equally likely to oviposit when offered an opportunity for one night at weekly intervals. Fourth, the duration of the juvenile stages from oviposition to emergence as an adult is 10–12 days. Previous data [22] showed that the distribution of the duration of juvenile stages differs between transgenic and non-transgenic pupae with transgenic larvae developing to the pupa stage more slowly (Figure 1B). For each individual, the precise duration is drawn from the relevant empirical distribution shown in Figure 1B, after which the individual becomes an adult with adult age zero. Finally, sufficient eggs are harvested in any given week so that at least 200 adults will emerge in the subsequent week and, of these, 200 are returned to the adult pool. Note that according to our model the transgene reduces fitness only through the cost of complete male sterility and through a somewhat prolonged juvenile development.

**Figure 2:**
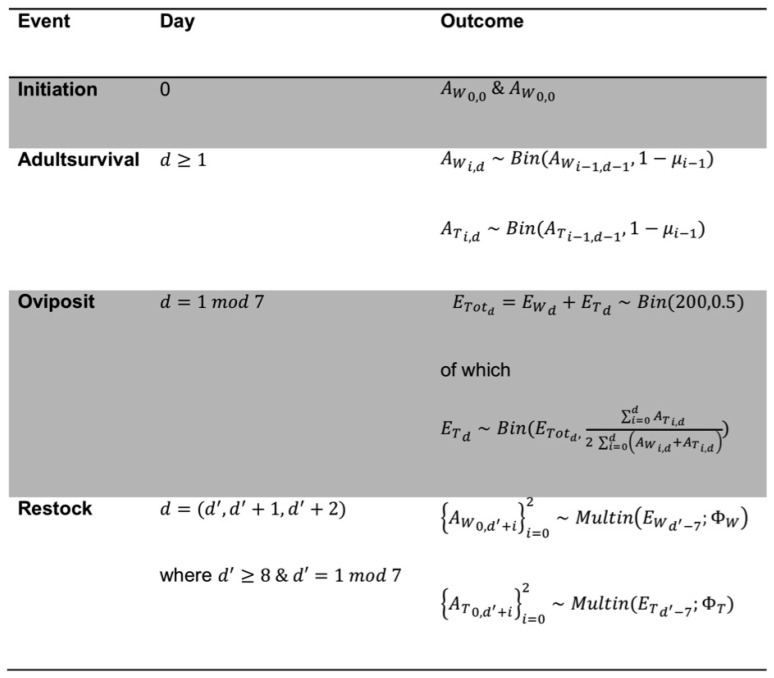
The simulation algorithm. The females in the population on day d are described by the variables $\left({A_{W}}_{i,d},{A_{T}}_{i,d}\right)$ (adults, *i* denotes age) and $\left({E_{W}}_{d},{E_{T}}_{d}\right)$ (juveniles), where the subscript W or T denotes wildtype or transgenic. The parameter $\mu_{i}$ is the probability an adult female dies at age *i* (derived from the survivorship function shown in Figure 1A), and $\Phi_{W}$ and $\Phi_{T}$ are the probability vectors of pupation after one, two, or three days (Figure 1B). The probability that a given egg is transgenic is half the fraction of transgenic individuals in the adult female population because all transgenic females are hemizygous (their fathers are non-transgenic), and Mendelian inheritance of the transgene has been confirmed experimentally [13,22].

Since we assume that transgenic males are always sterile, it follows that all viable eggs are fathered by wild-type males and the relative number of GM males has no bearing on the population genetic dynamics. Males are, thus, ignored by the model and the number of females emerging from each cohort is assumed to be half the cohort size. We denote ${A_{W}}_{i,d}$ and ${A_{T}}_{i,d}$ to be the numbers of wild-type and transgenic adult female mosquitoes of age *i* (days) on day *d* of the experiment, and ${E_{W}}_{d}$ and ${E_{T}}_{d}$ to be the numbers of viable female eggs harvested (Figure 2).

The simulation algorithm tracks these variables on a day-by-day basis by iterating the randomizations given in Figure 2. For each experimental treatment given by the initial fraction of transgenic adults, we ran 1000 replicates of the simulation in the software Mathematica (v. 10; Wolfram Research Ltd., Long Hanborough, UK), which was deemed a sufficient number of replicates to fully sample the expected distribution of transgene proportion at each time step.

To estimate the realized genetic load that the transgene actually imposed on life-history parameters based on the outcome of the experiments, we inferred a posterior distribution for the best-fitting model parameters by the following procedure, ABC rejection sampler [24,25]. First, the algorithm drew values for two parameters (L_e_, load on egg batch size, and L_l_, load on adult longevity) from a prior distribution. Second, a time series of transgene proportion was simulated using the model with the given parameters, for each of the initial conditions applicable to the transgenic strain. Finally, the parameter set was ‘accepted’ if the sum of square distances between the simulated and actual time-series was below a tolerance threshold. The estimated joint posterior distribution is the set of all accepted parameter sets, and we calculated the overall load on genetic fitness by the product 1 − (1 − L_e_)(1 − L_l_). We used a uniform prior distribution by selecting the load on egg batch size from U (0, 1) and load on longevity from U (0, 0.7) (the restriction to less than 0.7 prevents the possibility that all females die in the first week of the simulation). We used a tolerance level equal to the 1% quantile of the sum of square distance samples, which was judged to give a satisfactory balance between the number of posterior samples (which increases with tolerance) and the concentration of posterior density (which reduces with tolerance [25]). This produced 100 posterior points out of 10,000 prior samples.

### 2.2. Laboratory Experiments

The experimental population study was performed at the insectary of the Genomics and Genetics Section, Università degli Studi di Perugia, Italy. The populations of adult mosquitoes were maintained in custom-built plastic cages made from polypropylene household storage containers (30 × 50 × 28 cm, L × W × H) that had been modified with access sleeves and window screen ventilation panels. Temperature and relative humidity were held at 27 °C and 70%, respectively. Adults were provided with 10% sucrose plus 0.1% methylparaben (both *w*/*v*) as a preservative [23] continuously from hanging inverted sugar feeders.

#### 2.2.1. Mosquito Strains

The WT strain was an *A. gambiae* line, G3, which has been maintained in the laboratory for approximately 30 years and originated from a wild-type collection as stock number MRA-112 [26] obtained from the Malaria Research and Reference Reagent Resource Center. Due to the male sexual sterility conferred by the transgene, transgenic females are selected every generation and backcrossed to G3 males in order to maintain the strain and to produce the mosquitoes for the initial population seeding. This backcrossing scheme results in G3 and transgenic strains that are highly similar with the exception of the transgene itself. The GM sexually sterile male strains of *A. gambiae* were β2Ppo1 and β2Ppo2 [13], which have been renamed “Ag(DSM)1” and “Ag(DSM)2”, respectively, since their original publication by the Target Malaria project (www.targetmalaria.org), which developed them. We will use the newer terminology throughout. The strains were developed from the G3 strain and are marked with the fluorescent marker *3XP3DsRed* and *beta2-tubulinGFP*, the latter of which is fused to the sterility effector nuclease, *I-Ppo*I. The *beta2-tubulin* promoter causes expression of the GFP/nuclease fusion protein in the testes.

#### 2.2.2. Study Protocol

Overlapping generation cage populations were initiated using mixtures of virgin hemizygous transgenic Ag(DSM)1 or Ag(DSM)2 females and WT virgin females and males 2–4 days of age by one initial introduction. We conducted three preliminary experiments with the Ag(DSM)1 line at three release proportions: 100% (200 hemizygous GM females and 200 WT males), 50% (100 GM females, 100 WT females and 200 WT males), and 20% (40 GM females, 160 WT females and 200 WT males). Based on experience with Ag(DSM)1, we conducted three replicates of each of two release proportions with the Ag(DSM)2 line, 50% and 20%. The 100% release proportion performed with Ag(DSM)1 was deemed to provide little information that could not be achieved by the 50% release and was discontinued for Ag(DSM)2.

We blood-fed females every seven days for 30 min. with defibrinated and heparinized sterile cow blood that was kept frozen until use (Allevamento Blood di Fiastra Maddalena, Teramo, Italy) using a Hemotek membrane feeder (Discovery Workshops, Accrington, UK) covered with stretched Parafilm “M” (VWR, Milan, Italy) membranes. Eggs from each treatment were collected, washed with 1% household bleach to inhibit microsporidia infection and kept overnight on a damp filter paper disk placed in a covered polystyrene disposable Petri dish. The following day, all eggs were placed in a single tray with 500 mL of water for hatching and provided 5 mL of 2% *w*/*v* larval diet [27]. The following day, larvae were apportioned into three trays, each containing about 250 larvae using a standard rearing protocol [28] until they reached the 3rd and 4th instars when a sample of 200 larvae were arbitrarily selected by pouring, followed by counting and screening for fluorescence. The *3XP3DsRed* transgene fluorescent marker was detected using an Evos F1 microscope (Life Technologies Italia, Monza, Italy) to distinguish all transgenic individuals. GM and WT larvae were separated and transferred into two different trays and the sexes of pupae that formed were counted and separated by observing their genital morphology and reintroduced into the cage from which they originated. Mortality that occurred between placing pupae in the cages and eclosion was recorded. Populations in the cages were maintained by restocking all pupae regardless of the day of pupation in that cohort. The extant adults from the previous cohorts of adults were not removed, therefore overlapping, rather than discrete, generations were continuously maintained in the same cage after inception. The populations were assumed to have a stable age distribution determined by the rate of introduction of pupae and the realized longevity in these cages.

The experimental plan was to terminate each cage when no fluorescent offspring were detected in at least two consecutive ovipositions though somewhat arbitrarily some cages were terminated after one negative ovipositon and others were maintained longer. The primary outcome of interest was the frequency of the transgene among L4s as a function of week from the start of the experiment, grouped by the transgene initial proportion.

The model made no assumptions that there were any differences in the proportions of transgenic progeny, hatching rate, or number of eggs in successive ovipositions by the same females. An experiment was performed to determine this. Transgenic Ag(DSM)2 females were blood-fed and allowed to oviposit several times successively to determine whether the number of eggs differed by oviposition sequence and the total number of ovipositions.

### 2.3. Statistical Analysis

The data arising from the experiments are a weekly time series with sampling error (proportion transformed can rise and fall) and standard survival analyses could not be used. To allow for the temporal pseudoreplication arising from measurement of sequentially-linked cohorts (replicates), mixed effects models were used to identify whether the observations differed from the model predictions. The fixed effect was a factor of three levels: Ag(DSM)1, Ag(DSM)2, and Model, except in the case of the 100% experiment, which was only performed with one line, the Ag(DSM)1 line. Random effects were used to indicate that week represented the pseudoreplication within individual trajectories. Comparisons were made until extinction was observed in the experimental data. Analyses were performed in R 3.2.3 with the non-linear mixed-effects package “nlme” [29].

Comparisons of sex ratios and pupal mortality used counts summed over each experimental replicate. In these cases, binomial (or quasibinomial where data were overdispersed) generalized linear models were used to identify important variation in the proportion female and the proportion alive. Model simplification used chi-squared tests for binomial models and F tests for quasibinomial models. The data analyzed were, as appropriate, the number of females and the number of males and as well as the number of pupae dead and alive each bound together pair-wise in a weighted variable. WT survival and sex ratio were analyzed as a function of the GM strain that was used for the initial seeding and the treatment (% seeding). GM survival and sex ratio were analyzed as a function of strain and treatment. Where parameter estimates are given, means are presented with their standard error (SE).

The investigation of the proportion of transgenic individuals by oviposition following successive feeding uses Poisson family models for counts and a binomial family for proportions. The number of eggs laid (count), hatching rate (proportion), and the proportion transgenic were analyzed as a function of the sequential feeding opportunities. Analyses were performed in R 3.2.3 [30].

## 3. Results

**Figure 3:**
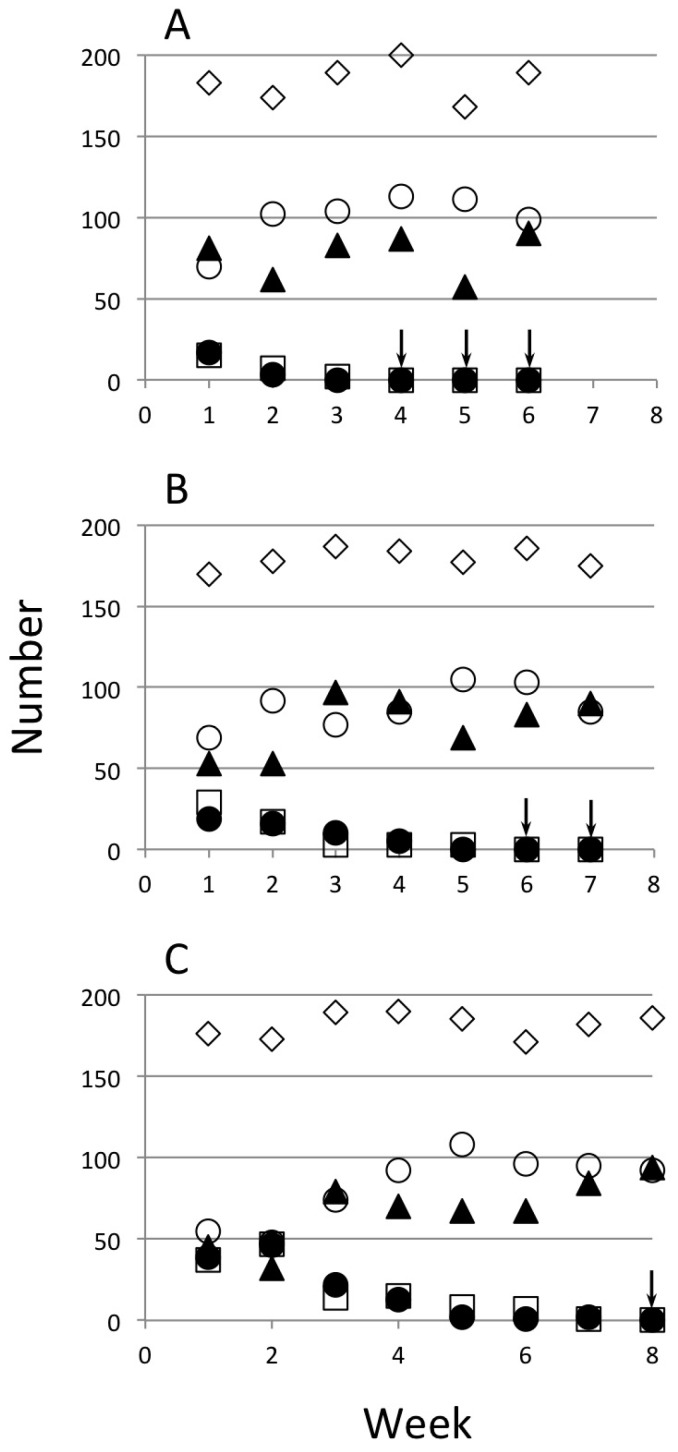
Restocking of Ag(DSM)1 cages. The actual numbers of pupae returned each week and the totals are represented. Three frequencies of seeding of hemizygous transgenic females were made: 20%, 50%, and 100% (**A**–**C**, respectively). Transgenic males (⚫), transgenic females (☐), non-trangenic males (▲), non-transgenic females (⚪), and the total (◇). Arrows indicate weeks in which no GM mosquitoes were detected.

**Figure 4:**
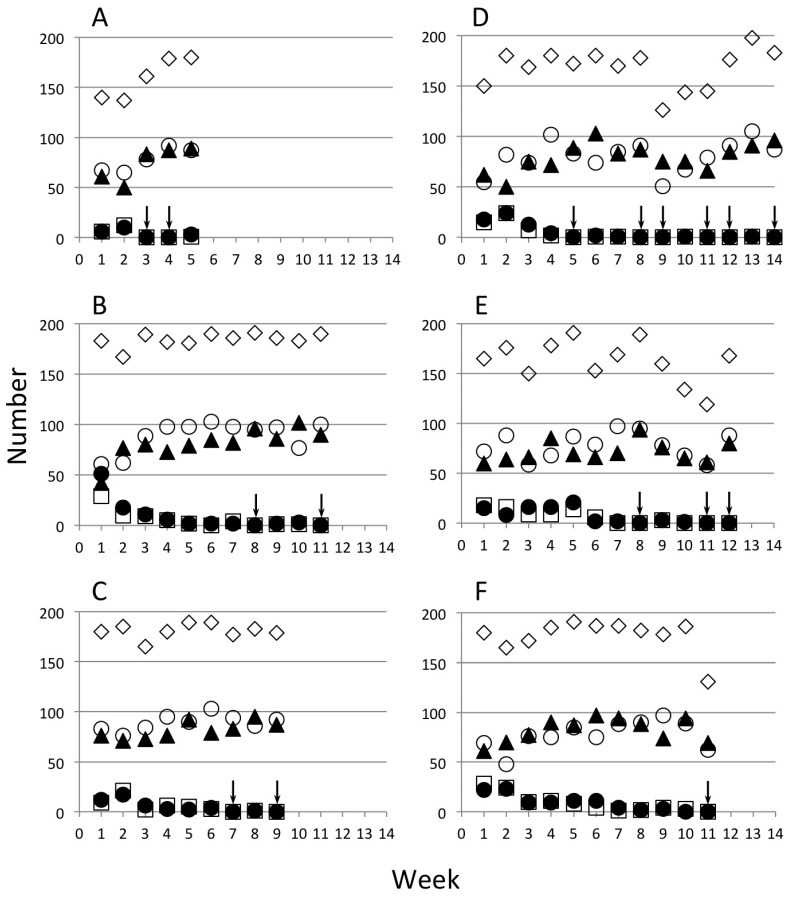
Restocking of Ag(DSM)2 cages. The actual numbers of pupae returned each week and the totals are represented. Two frequencies of seeding of hemizygous transgenic females were made: 20% (**A**–**C**), 50% (**D**–**F**). Transgenic males (⚫), transgenic females (☐), non-trangenic males (▲), non-transgenic females (⚪), and the total (◇). Arrows indicate weeks in which no GM mosquitoes were detected.

In all cohorts, 200 larvae were sampled and their transgenic status and sex were determined. The actual numbers restocked in the cages were lower than 200 due to mortality between larval examination and adult emergence. The number of GM and WT females and males that were restocked in cages of Ag(DSM)1 at three initial proportions of females (Figure 3) and the three trials of Ag(DSM)2 at two initial proportions (Figure 4).

Within the WT mosquitoes, the female proportion did not vary between seeding densities (*F* = 2.13, d.f. = 5,7, *p* = 0.21), but this measure differs between the experiments (*F* = 10.93, d.f. = 7,8, *p* = 0.013). In the earlier experiments with Ag(DSM)1, the WT population was slightly female-biased (0.55 ± 0.01), which was not the case in the later experiment with Ag(DSM)2 (0.51 ± 0.01). The female proportion in the GM strain was as expected (0.49 ± 0.02) and did not vary with the seeding density (*F* = 0.40, d.f. = 5,7, *p* = 0.69), there was also no difference between the strains (*F* = 3.11, d.f. = 7,8, *p* = 0.12).

The development rate from hatching to the first day of pupation for the model was fixed at six days. The mode (17 out of 33 points) of the observed number of days between hatching and first pupae was six days ($\bar{x}$ = 6.21, SE = ±0.15). The model assumed that pupae would form within three days thereafter. All larvae that pupated from the larvae screened were collected, and the observed number of days for the development of all pupae varied from 3–7 days with a mode of 4 (18 out of 33 points, $\bar{x}$ = 4.36, SE = ±0.14). Overall, 87% of the pupae formed in the first three days.

Mean (±SE) GM pupal mortality (0.037 ± 0.006) did not vary significantly between strains (*F* = 0.38, d.f. = 13,14, *p* = 0.55), nor with seeding density (*F* = 0.15, d.f. = 14,16, *p* = 0.87), nor by sex (*F* = 0.57, d.f. = 16,17, *p* = 0.46). WT pupal mortality did not vary between treatments (*F* = 0.38, d.f. = 13,15, *p* = 0.69) nor by sex (*F* = 0.32, d.f. = 15,16, *p* = 0.58), but there was an apparent effect of seeding GM strain used. In the experiments with Ag(DSM)1, WT mortality was lower than in the experiments with Ag(DSM)2 (${\bar{x}}_{1}$ = 0.016 ± 0.03 and ${\bar{x}}_{2}$ = 0.043 ± 0.006, *F* = 10.17, d.f. = 16,17, *p* = 0.006).

In observations of ovipositions resulting from successive feeding opportunities, of the 18 females investigated, 12 laid at the first feed, nine at the second, and four at the third. Variation in the number of eggs laid by each female in this investigation was normally distributed and not a significant factor (*F* = 2.31, d.f. = 8,22, *p* = 0.12), nor did the number of eggs laid per female at subsequent feeds differ from the first (*F* = 0.13, d.f. = 8,10, *p* = 0.88). Hatching rate (0.89 ± 0.01) was normally distributed and did not vary significantly among eggs laid by different females (*F* = 0.80, d.f. = 8,22, *p* = 0.66), neither did this measure vary between the ovipositions resulting from successive feeds (*F* = 1.75, d.f. = 8,10, *p* = 0.23). The proportion of GM progeny was monitored over successive ovipositions for four of the females (n = 711 WT, 704 GM). This measure was 1:1, as expected (0.50 ± 0.1), and did not vary between the females (χ^2^ = 5.65, d.f. = 3, *p* = 0.13) nor as a function of oviposition number (χ^2^ = 0.18, d.f. = 1, *p* = 0.68).

**Figure 5:**
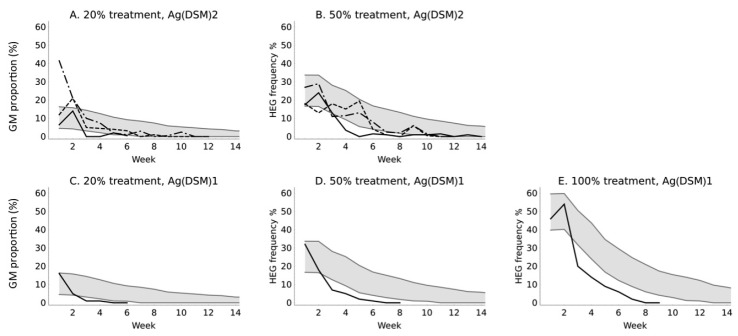
Model predictions and observations of transgene frequency in unselected populations. The modeled frequency expected of the transgene in the experimental populations when seeded at different frequencies and the observations for either GMM line. The gray bands show the 95% central quantile intervals of the model trajectories. The dashed and solid black lines represent the experimental results for three replicates of two treatments with Ag(DSM)2 (**A**,**B**) and the single experiments at three treatment levels with Ag(DSM)1 (**C**–**E**).

The primary response variable of interest in this study was the frequency of the transgene in populations not under deliberate artificial selection with regard to the transgene. In all comparisons, the observed data depart from the model predictions (Table 1). This is mostly because the decline in the proportion transformed was more rapid than predicted by the model (Figure 5). However, in one case in one cage we observed an anomalously high frequency of transgenic individuals in the Ag(DSM)2 20% experiment. One explanation is that the transgenic females were more mature and bloodfed more avidly than the wild-type when the cage was initiated, but it is impossible to be certain. This effect would be most evident when the cages were initiated since populations with a greater degree of age-mixing would exist thereafter.

Since the rate of decline in the transgene proportion was more rapid than expected, a post hoc analysis of the observed fitness of the two strains was conducted. The predictive model assumed the main effect of each transgene on life-time reproduction is male sterility, giving a genetic fitness of 0.5 for both strains. We determined that the best fit value for fitness was 31.5% (26.8%–37.1% with 95% probability) and 20.5% (9.0%–28.1%) lower than this for strains Ag(DSM)1 and Ag(DSM)2, respectively. This indicated that additional causes of fitness reduction were occurring, beyond the male sexual sterility factor and the prolonged larval stage.

## 4. Discussion

With regard to the measured life-history parameters that were assumed by the model the assumptions used in the model were similar to previous observations [13,22]. The proportion of transgenics conformed to the expected 1:1 ratio among progeny of the individual transgenic females tested; the sex ratio did not differ greatly from a 1:1 and only in the case of the Ag(DSM)1 experiments. The duration of pupa formation was somewhat longer than used in the model. Regardless, in these experiments, we observed a 20%–30% faster decline in the frequency of the transgene than was expected. In the context of these studies, risk, the observation of faster decline than expected makes the mechanisms that cause this of less concern than if the decline had been slower than expected. However, there are several biological characteristics of the mosquitoes that have not been studied that might explain this and that would be interesting to pursue: lower GM female adult survival relative to WT, reduced mating by transgenic females, or differences in fecundity. No differences in the number of eggs laid per female that oviposited between Ag(DSM)1, Ag(DSM)2 and the WT was observed previously (Supplementary Table S1), but it is still possible that the GM females do not blood-feed or fail to oviposit at all at a higher rate than the WT females. It is also possible that higher rates of decline than we modelled could have occurred due to some unknown factor causing underdominance of the hemizygous GM individuals. Specific observation of these factors should allow further parameterization of models to account for these effects if they are found to occur.

It is possible that retarded larval development [22] and reduced mating competitiveness [22,31] result from effects on vigor attributable to expression of the nuclease in the testes but not likely from the insertion of a transgene, per se. The two strains that carry the same transgene in different chromosomal locations both show these effects, whereas three others that carry the same transgene with the exception of a single amino acid modification of the nuclease to decrease its persistence [21] do not (unpublished data [32]). Testes-specific expression using the *beta2-tubulin* promoter used in the transgenic mosquitoes in this study was determined based on GFP expression, which is observed only in the testes, but expression in other tissues has not been studied.

The observations were made under highly simplified conditions in comparison to those that mosquitoes experience in the field. Whereas our observations were made in highly controlled and uniform conditions, variable light, temperature, and humidity, landscape heterogeneity, and species interactions are natural influences that can potentially affect transgene persistence. The study that is reported here should be considered a first approximation of transgene persistence. If one assumes that the stresses in natural environments are greater than in the laboratory, one might expect that reduced vigor of the transgenic individuals would diminish their persistence even more so than what we observed under controlled laboratory conditions.

Of the two methods for determining the fitness of transgenes, continuous populations vs. discrete generations, the former provides several advantages that may result in more realistic estimates of fitness. Among the factors that can be captured are the effects of age on mating frequency, blood-feeding likelihood and fecundity and interactions between adults of different ages. Continuously maintained populations are not simpler to maintain as the adults are kept in the same cage which must be kept clean and intact for a longer period of time, but the difficulties are not insurmountable. Continuously breeding overlapping populations also offer the possibility of direct competition in which unidentified life-history characteristics can affect the outcome. A similar design could also be used to compare different transgenes, for example, genetic markers or docking sites in mixed populations to directly measure their relative fitness in identical environments. Since the various types being compared are exposed to the same temperature and humidity and handling regimes, the experimental conditions are as closely matched as possible, thus minimizing sources of variation due to factors such as separate cages, handling, and their location.

The value of models in risk assessment rests on the accuracy of their predictions. In this case we have a model with clearly-defined frequency distribution ranges against which we compared experimental observations. Risk is a measure of likelihood and consequence. The model tells us something about likelihood but nothing about consequence, other than that the genetic material disappears at a particular rate. It is not an indicator of harm, but in this case it certainly shows reversion to WT, which is probably an indication of lack of harm. Models that overestimate efficacy of the intervention and underestimate risk factors can mislead risk assessment decisions. Therefore, experiments like these that measure model predictions provide useful starting points for evaluating models. These data demonstrate that this transgene is likely to disappear quickly in the environment. The model, coupled with evidence from mark-release-recapture studies, could provide a basis for a field-sampling plan to demonstrate non-persistence under various seasonal conditions in an application for field release approval.

The strains tested here are for contained use in African settings. They are expected to be of minimal environmental impact and are to be used for capacity building in Africa, rather than for control purposes. The increased capacity is being built in preparation for handling and releasing strains of greater potential as a malaria vector control technology. To the best of our knowledge no laboratory in Africa has obtained permits for transgenic mosquitoes in containment. This study contributes to the risk assessment methodology for these strains as in the unlikely event that there could be accidental releases from containment, it shows that the transgene is predicted to disappear rapidly from the environment.

## 5. Conclusions

These models and experiments provide an example of an assessment of the outcome of a transgene persisting from a mixed population of wild and transgenic mosquitoes in the course of contained use studies or in an approved release. These comparisons are only one part of such an assessment, but we believe that this demonstration of rapid decline in frequency indicates that any risk that depends upon this characteristic is low, and that persistence is even lower than predicted. It does not imply that any persistence of the transgene would result in harm, but should help to limit that possible concern.

## Figures and Tables

**Table 1:** The log ratio and likelihood of the experimental data conforming to the predictions of the disappearance model as a function of the initial release density and transgenic line.

Strain	Initial Release Level	L Ratio	d.f.	*p*
Ag(DSM)1	100%	34.47	5,6	<0.001
	50%	21.07	6,7	<0.001
	20%	4.62	6,7	<0.05
Ag(DSM)2	50%	32.93	6,7	<0.001
	20%	9.39	6,7	<0.01

## Supplementary Materials

The following are available online at www.mdpi.com/2075-4450/7/3/47/s1, Table S1: Number of eggs oviposited by females according to transgenic status and mate in Excel file “Supporting Egg Data”.
